# The relationship between core muscle endurance and functional movement screen scores in females with lumbar hyperlordosis: a cross-sectional study

**DOI:** 10.1186/s13102-022-00567-2

**Published:** 2022-10-13

**Authors:** Eiva Fallahasady, Nafise Rahmanloo, Foad Seidi, Reza Rajabi, Mohammad Bayattork

**Affiliations:** 1 Health and Sports Medicine Department, Faculty of Physical Education and Sport Sciences, University of Tehran, Tehran, Iran; 2 Health and Sports Medicine Department, Faculty of Physical Education and Sport Sciences, Alborz Campus, University of Tehran, Tehran, Iran; 3 Sport Sciences and Physical Education, Faculty of Humanities Science, University of Hormozgan, Bandar Abbas, Iran

**Keywords:** FMS, Core strength, Postural disorder, Stability, Lordosis

## Abstract

**Background:**

Core muscle endurance is essential for proper movement and lower extremity injury prevention. In addition, the Functional Movement Screen (FMS) score is a tool to assess body movement patterns to predict the risk of injury. Although various researches have investigated the relationship between the core muscle endurance and the FMS score, no study has ever assessed the effect of postural deformity on the FMS score. This study investigates the relationship between core muscle endurance and FMS scores in females with lumbar hyperlordosis.

**Methods:**

42 healthy females aged 24.03 ± 4.4 years with hyperlordosis ( > = 45/66 degrees) participated in this study. Core muscle endurance was assessed by the McGill stability test. Correlations were evaluated between the FMS score, McGill test, and lordosis angle using spearman correlation coefficients (p ≤ 0.05).

**Results:**

Most individual FMS scores were not correlated with the McGill test except stability trunk push up. Also, lordosis angle was not correlated with the FMS composite score (r=-0.077; p = 0.631), while it was negatively correlated with the McGill test (r=-0.650; p = 0.000).

**Conclusion:**

The lack of correlation between the FMS score and the McGill test implies that one’s level of core endurance may not influence their functional movement patterns. In contrast, the lumbar lordosis angle might impact one’s core muscle endurance but not their functional movement patterns.

## Background

Lumbopelvic stability is the ability to maintain balance and control the motion and position of the lumbar spine and pelvis relative to a neutral position during kinetic chain activities[1]. The deficit in lumbopelvic stability may lead to neuromuscular impairments, which is common among people with low back pain[2]. According to the literature, deep spine stabilizers, particularly the Transverse Abdominal muscle (TrA)( the primary stabilizer due to the direction of its fibers[3]), the pelvic floor muscles, and the Lumbar Multifidus muscle (LM), are all responsible for the integrity, proper functioning, and the correct level of stability in the spine [4] Even though co-contraction of the trunk extensors and flexors increase the stability of the spine[5], core stabilization of the lumbar spine has been highlighted as one of the critical factors for proper movement of the lower extremities[6], which provides spinal stability and prevents lumbopelvic region injury[7]. As mentioned, lumbopelvic stability occurs by core muscles, which is called core muscle endurance. Therefore, many practitioners have developed practical training programs for patients with lumbopelvic instability focusing on core muscles, specifically TrA and LM [1].

On the other hand, lumbopelvic stability could be associated with postural alignment and might be disturbed by changing spinal postural alignment, such as lumbar hyperlordosis [8]. Lumbar lordosis is the inward curvature in the lumbar spine formed by the sum of vertebral discs and bodies wedge angles, and it might be changed by the intervertebral discs degeneration and the vertebral bodies compression[9]. Dorsal wedging of the vertebral bodies and discs will lead to hyperlordosis [10]. Lumbar hyperlordosis has been considered as the major cause of facet pain, postural pain, and radiculopathy [11]. In addition, a greater lordosis angle is believed to be one of the main risk factors for developing ventral slippage and spondylolysis of the affected vertebra[12]. Many studies have suggested that lumbar lordosis, pelvic tilt degree, and abdominal muscle function are interrelated. In other words, hip instability and weakness in abdominal muscles coexist, leading to increased psoas muscle activity and thus anterior pelvic tilt and the hyperlordotic posture finally[13]. Changes in lumbopelvic alignment are significantly associated with biomechanical and tissue changes in a patient with hyperlordosis. Subsequently, it disrupts lumbopelvic stability and rhythm due to deformity and pelvic ring distortion[14]. Moreover, based on the chain reaction theory, Janda [14]argued that due to the interactions of the neural, skeletal and muscular systems, dysfunction of muscles or joints due to postural malalignments may influence movement patterns and function.

The Functional Movement Screen (FMS) is an assessment tool that has been designed to measure trunk and core strength as well as fundamental movement patterns in a series of physical activities (deep squat, hurdle step, in-line lunge, shoulder mobility, active straight leg raise, trunk stability push-up, rotary stability)[15–17]. The FMS can identify poor quality movement patterns, any dysfunction, and asymmetry in the body[16]. It is mainly used to predict the risk of future injury and establish a movement baseline after treatment, rehabilitation, or performance training for active adults [15–17]. Since poor posture can affect joints, bony structures, and soft tissues, resulting in musculoskeletal disorder [18], therefore, it seems logical to use the FMS score as a postural assessment tool. Among several methods of core muscle assessment, the McGill test is the most reliable isometric test to evaluate core muscle endurance and stability[19] which is used in previous studies in a different age of people with different levels of physical activity[20–22]. As mentioned above, there seems to be a direct relationship between posture and lumbopelvic stability [8]. Granata et al. [8] have found that spinal stability is affected by posture and trunk muscle recruitment patterns. In various studies, the importance of lumbopelvic stability and proximal stability has been identified as an essential element for proper movement in the lower extremities and distal mobility[6, 23, 24] .In this regard, Cook et al.[16, 17] have reported a negative correlation between FMS score and movement deficit. Also, Okada et al. [25] have found no significant correlations between core stability and FMS score in healthy adults. On the other hand, Soltanidoost et al.[26] have indicated a negative correlation between pain and FMS and a positive relationship between the FMS, trunk muscle endurance, and dynamic balance in military personnel with non-specific low back pain. In addition, Bagherian et al. [27] have reported an enhancement in functional movement pattern and dynamic postural control after eight weeks of core stability training program. Mitchel et al. [28] have also indicated a positive correlation between core strength and the FMS results and no correlation between static posture and FMS Score in students aged 8 and 11 years. Finally, Anderson et al. [29] have found a stronger correlation between core muscle endurance and movement capacity in women than men. Therefore, on one hand, core strength correlated with FMS and hyperlordosis angle and on the other hand, lumbar lordosis correlated with core muscle endurance and might lead to movement deficit which FMS could be affected by these deficits.

According to the best of our knowledge, researchers have not investigated the effect of spinal deformities on movement patterns and core muscle endurance. It would be helpful to understand whether the result of the FMS and McGill test is affected by the spinal deformity. Therefore, we hypothesized that individuals with higher lordosis angles would acquire lower scores in the FMS and McGill tests, both of which are used as an injury prediction tool[30, 31]. Such a relationship might be acceptable as spinal deformity influence movement pattern and the musculoskeletal system. Thus, the primary purpose of the current study was to determine whether there existed a relationship between FMS score and core muscle endurance in young females with lumbar hyperlordosis. The secondary purpose was to investigate the relationship between the FMS score and lordosis angle and the McGill test and lordosis angle.

## Methods

Study Design: The current study was cross-sectional and assessed whether functional movement and core muscle endurance are associated with lordosis angle in young females. All participants were received oral and written testing procedures and were required to sign an informed consent. The reporting of this study conforms to the guideline ‘Strengthening the Reporting of Observational Studies in Epidemiology’ (STROBE). The study was approved by the Human Subjects’ Review Boards of University of Tehran and was accomplished as part of a master’s thesis in Health and Sport Medicine Department.

Participants: Forty-two healthy young females (age: 24.03 ± 4.4year, height: 161.6 ± 0.05 cm, and weight: 56.66 ± 7.97 kg) with lumbar hyperlordosis deformity ( > = 45.66 degrees) were chosen from the Annual Screening Program conducted by the University of Tehran in which 700 female students participated voluntarily, and 350 of them were identified as hyperlordotic. All junior students of the University of Tehran from different faculties and departments participate in the health screening program each year. None of the subjects were studied in the faculty of sports science. From these samples, only 50 participants were included in this study based on the following criteria. Inclusion criteria were being older than 18 years and having a lumbar lordosis angle greater than 45 as measured by a flexible ruler according to the procedure described by Seidi et al. [32]. Flexible ruler is a portable and low-cost method of measuring lumbar lordosis angle with high validity (0.91) and reliability (≥ 0.82)[33, 34]. Participants were excluded from the research process if they had any experience of pathological signs, ankle injury or instability, low back pain, extremity injuries or surgeries, or any problem in their spine, shoulder, pelvic girdle, or musculoskeletal disorder, mainly upper cross syndrome, participating in professional physical activity and were pregnant. Eight participants were excluded due to the pain feeling while performing the test.

Procedures: After signing the informed consent and collecting anthropometric data (height, weight), each participant completed a 10 min warm-up, consisting of two min running and five min whole-body exercises, and three min flexibility work-out. The participants were instructed how to conduct the protocol by examiner demonstration and verbal explanation. Then, they performed the tests one by one immediately. They conducted the McGill core muscle test and then the FMS after a five min rest.

The McGill test: McGill test has been used to assess core muscle endurance [20, 22] which is composed of trunk flexor (sit-up position withholding the back in 60° from the floor), trunk extensor (prone position with upper limb above the Anterior Iliac Spines (ASIS) hanging off the table), and right and left lateral trunk musculature tests (left or right side-lying on the floor with 90° bent elbow and positioned under the shoulder with raised off pelvis)[35]. The participants were encouraged to maintain the isometric postures instructed by the examiner completely for each test position as long as possible, and they did the test only once. The length of time they could hold the correct position for each posture was recorded, and the results of four subtests were added to get an overall score[35]. The McGill test has shown excellent reliability( ICC = 0.97-0.99)[19].

The FMS: The FMS comprises seven movement patterns: deep overhead squat, in-line lunge, hurdle step, active straight leg raise, trunk stability push-up, shoulder mobility, and rotary stability. The participants performed it barefoot. For further analysis, all subtests were recorded using two cameras, one at the sagittal plane and the other at a frontal plane. All participants conducted three trials of each movement pattern scored according to the FMS manual by Cook [15], which scored from zero to three. If each subtest was performed perfectly, scored three; if the compensation movement was occurred to complete the subtest, scored two; if the subtest was not completed, scored one and scored zero if the pain was experienced. The highest score of the three trials was recorded and used for subsequent analysis. However, the lower of the two scores was recorded where the movement pattern was performed separately on the left and right sides (i.e., lunge, active straight leg raise, hurdle step, shoulder mobility, and rotary stability). The FMS has indicated excellent interrater reliability [36] and moderate to strong intrarater reliability (ICC = 0.77-0.94) [37].

Statistical Analysis: The data were analyzed with descriptive and inferential statistics using SPSS Version 24.0 (SPSS Inc., Chicago, IL, USA). Pearson Product Moment Correlations were performed to examine the relationship between the composite FMS score and the McGill test, and the McGill test with lordosis angle; whereas, Spearman Coefficient Correlation was conducted to determine the relationship between the subtests of both the FMS and core muscle endurance and the individual FMS score with lordosis angle. To describe the strength of the correlation, the following scale was used for the absolute value of the correlation coefficient (r): strong relationship (0.5 ≤ r ≤ 1.0), moderate relationship (0.3 ≤ r < 0.5), and weak relationship (r < 0.3)[38]. The α-level was set at p ≤ 0/05.

## Result

The mean and standard deviation for the composite FMS score, each individual McGill test, and lordosis angle are presented in Table 1. The median of all individual FMS scores was 2 except for push-up (= 1). The percentage of scoring distribution for each individual FMS subtest according to the scoring system was as follows: deep overhead squat (0 = 4.9%, 1 = 0.8%,2 = 85.4%); in-line lunge (1 = 4.9%, 2 = 90.2%, 3 = 4.9%); step hurdle (1 = 4.9%, 2 = 90.2%, 3 = 4.9%); active straight leg raise (1 = 2.4%, 2 = 53.7%, 3 = 43.9%); shoulder mobility (1 = 9.8%, 2 = 41.5%, 3 = 48.8%); trunk stability push-up (0 = 4.9%, 1 = 56.1%, 2 = 29.3%, 3 = 9.8%). It should be noted that none of the participants scored 3 in deep overhead squat.

**Table 1:** Descriptive statistic for the composite FMS score, McGill test score, and lordosis angle

	TFMS	TMG	LSP	RSP	EXT	FLX	Lordosis	
Mean	13.61	193.34	33.37	31.81	65.59	62.56	57.53	
Std. Deviation	1.430	68.36	16.05	14.38	32.93	28.79	4.99	

*Note*. TFMS: Total Functional Movement Screening Score; TMG: Total McGill Score; LSP: McGill Left Side Plank; RSP: McGill Right Side Plank; EXT: McGill Extensor; FLX: McGill Flexor

**Table 2:** Correlation between each FMS score and McGill test

	TMG(SD)	RSP(SD)	LSP(SD)	EXT(SD)	FLX(SD)
TFMS	0.130(0.417)	0.102(0.527)	0.046(0.776)	0.251(0.113)	-0.041(0.799)
DS	0.075(0.640)	0.045(0.779)	0.53(0.743)	0.071(0.657)	-0.056(0.727)
LILL	-0.246(0.122)	-0.178(0.266)	-0.057(0.722)	-0.214(0.180)	-0.260(0.101)
RILL	-0.119(0.459)	-0.020(0.902)	0.086(0.594)	-0.178(0.265)	-0.218(0.171)
LHS	0.045(0.781)	-0.086(0.593)	-0.041(0.800)	0.205(0.199)	-0.106(0.511)
RHS	-0.073(0.652)	-0.218(0.171)	-0.142(0.376)	0.112(0.485)	-0.284(0.072)
LSM	-0.166(0.301)	-0.162(0.311)	-0.115(0.474)	-0.091(0.570)	-0.79(0.623)
RSM	-0.146(0.363)	-0.084(0.602)	-0.223(0.161)	-0.043(0.792)	-0.050(0.757)
LSLR	0.028(0.861)	-0.150(0.350)	-0.118(0.461)	0.276(0.081)	-0.059(0.715)
RSLR	-0.047(0.770)	-0.210(0.187)	-0.180(0.261)	0.156(0.330)	-0.055(0.732)
LRS	0.095(0.556)	0.154(0.336)	0.213(0.181)	0.009(0.955)	-0.009(0.955)
RRS	0.036(0.822)	0.23(0.888)	0.100(0.535)	0.072(0.652)	-0.082(0.612)
PU	0.341(0.029)*	0.385(0.013)*	0.289(0.067)	0.221(0.165)	0.166(0.299)

*Note*.DS: Deep Squat; LILL: Left In-Line Lunge; RILL: Right In-Line Lunge; LHS: Left Hurdle Step; RHS: Right Hurdle Step; LSM: Left Shoulder Mobility; RSM: Right Shoulder Mobility; LSLR: Left Straight Leg Raise; RSLR: Right Straight Leg Raise; LRS: Left Rotate Stability; RRS: Right Rotate Stability; PU: Push Up.*p<0.05, **p<0.01

**Fig. 1:**
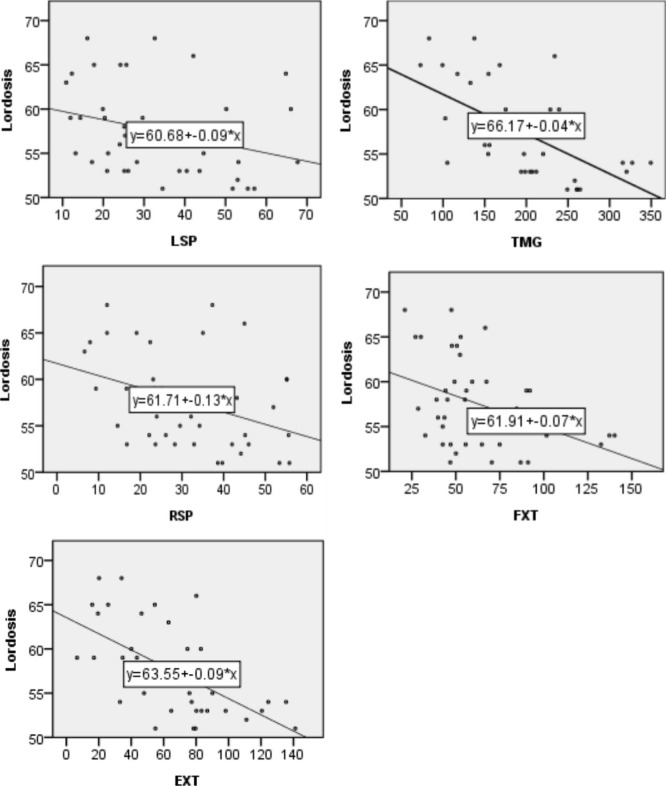
Correlation between lordosis and each subtest of McGill test, Lordosis and McGill left side plank LSP, Lordosis and total McGill test TMG, Lordosis and McGill right side plank RSP, Lordosis and McGill Flexor FLX, Lordosis and McGill Extensor EXT

The result of the analyses depicted no significant correlation between the FMS and the McGill test (r = 0.130, p = 0.417) (Table 2) as well as the FMS and lordosis angle (r= -0.113, p = 0.480) (Table 3). However, there was a significant correlation between the McGill test (TMG) and lordosis angle(r=-0.611, p=0.00) (Fig. 1). The individual FMS scores (deep overhead squat in-line lunge, step hurdle, active straight leg raise, shoulder mobility, trunk stability push-up) were also compared with each individual McGill test (Table 2). The only significant relationship found was between trunk stability push-up and core stability test. Trunk stability push-up was significantly correlated with right side plank (r = 0.385, p = 0.013) and the McGill composite score (r = 341, p = 0.029) (Table 2)

**Table 3:** Correlation between each FMS score and lordosis angle

	Lordosis
TFMS	-0.113(0.480)
DS	0.063(0.696)
LILL	0.069(0.668)
RILL	-0.020(0.902)
LHS	-0.093(0.565)
RHS	-0.053(0.742)
LSM	0.054(0.736)
RSM	0.66(0.680)
LSLR	0.038(0.815)
RSLR	0.089(0.578)
LRS	0.018(0.910)
RRS	0.148(0.356)
PU	-0.259(0.101)

*Note*.DS: Deep Squat; LILL: Left In-Line Lunge; RILL: Right In-Line Lunge; LHS: Left Hurdle Step; RHS: Right Hurdle Step; LSM: Left Shoulder Mobility; RSM: Right Shoulder Mobility; LSLR: Left Straight Leg Raise; RSLR: Right Straight Leg Raise; LRS: Left Rotate Stability; RRS: Right Rotate Stability; PU: Push Up

## Discussion

This study aimed to investigate the relationship between the FMS score and the McGill test in females with lumbar hyperlordosis, and; secondarily, the relationship between the FMS score and lordosis angle as well as core endurance with lordosis angle to determine the effect of this malalignment based on movement pattern and core muscle endurance. However, this hypothesis was not confirmed. The McGill test assessed core musclesto elicit isometric muscle endurance of trunk muscles[39], and functional movement patterns were measured using the FMS [15]. Our results showed no significant relationship between the composite FMS score and the McGill test score. In addition, although lordosis angle did not correlate with the FMS score, it had a strong correlation with the core endurance.

As previously mentioned, core muscle endurance is crucial to have better performance, especially in the lower body. However, neither the composite nor individual component FMS scores except the trunk stability push-up were significantly correlated to the McGill subtests. A previous study supports this result. Okada [25] believed that the lack of correlation between the FMS score and the McGill test result is odd despite the requirement of core stability to complete each task. Core muscles have an influential role in human movements and performance[24]. In contrast with the current study, this study was performed on healthy recreational athletes to compare the relationship between core stability, performance, and functional movement. Therefore, according to this result, the lack of the correlation between the FMS score and core muscle endurance implies that the functional movement pattern could be affected by other components such as body coordination, muscle strength, and recruitment patterns. For instance, a low score in the overhead squat is due to the weakness in the rectus femoris, tibialis anterior, and gastrocnemius[40]. The other reason can be the specificity of the tests. Like performance tests, the FMS is a repetitive-quick movement taking advantage of fast-twitch muscle fibers; while, the McGill test is an isometric muscle contraction for testing muscle endurance performed by slow-twitch muscle fibers[22]. Therefore, comparing these two tests might not be accurate and practical.

On the other hand, our results contrasted those of Mitchel et al.’s [28], who found a positive relationship between these two tests in healthy school children of Moldova. One reason might be the difference between the core muscle tests used in these two articles. In the study by Mitchel et al., only 3 out of 4 McGill subtests were utilized, including prone plank and left/right side plank. Another reason might be related to the scoring procedure. All their participants had to maintain correct posture for one minute to get the complete score, while our participants were scored based on the duration they could hold the correct position. Other possible components might have influenced the test results, such as body movement pattern, mobility and stability of lower extremity, muscle activation pattern, upper and lower extremity muscle endurance, and neuromuscular adaptation.

Interestingly, the FMS score for the trunk stability push-up correlated positively to the McGill test score and the right side plank. This means that better stability in the trunk results in a greater core endurance and a longer time for holding the right side plank. The trunk stability push-up is the ability to sustain a stable trunk to allow force transition throughout the body to the upper extremity[16]. The push-up and McGill tests are both performed in the sagittal plane and share similar body movement patterns and coordination. The right side plank was correlated with the push-up, which can be explained by considering the dominant leg of the participants. 39 out of 42 participants were right-legged, indicating that 92% of participants were dominant on the right side of their body during the test.

Several significant negative correlations were identified between core muscle endurance and lordosis angle in this study. It means that individuals with posture impairment have a lower core muscle endurance level. Previous studies have also indicated that individuals with lumbar hyperlordosis have less core stability due to the distortion of the lumbopelvic ring[14]; thus, the result of the present study confirms those of the prior research. In other words, muscles in charge of core stability, which create two force couples (hip flexors and lumbar extensors, hip extensor muscles and abdominal muscles), are responsible for maintaining the height of the pelvic and the imbalance of these muscles leads to changes in pelvic alignment and thus lumbar lordosis angle[41].

On the other hand, no significant correlation was found between all individual FMS scores and lordosis angle. The FMS score consists of muscle flexibility, coordination, endurance, strength, balance, and muscle movement[42], which might have impacted the test result despite compensatory movement due to lumbopelvic malalignment. This highlights the major finding of this study that the FMS score was not affected by hyperlordosis, and it is better to include the McGill test in addition to the FMS for further analysis in injury prevention.

In the end, we recommend that future researchers investigate the replicability and possibility of applying the present study’s findings to females and males of different age ranges, sports, and levels of physical activity. Second, future research can be conducted on larger sample sizes to validate the current study’s findings for broader populations. Third, other reliable and valid tests can be used to measure the core muscles components such as endurance, strength, and power (Sahrmann stability test, timed sit up, front abdominal power and abdominal side power).

## Conclusion

The FMS and the McGill test are the tools utilized by sports practitioners or trainers in rehabilitation, return to sport, predicting the risk of injury, and screening in different phases of training. This study identified neither correlation between these two tests nor between lordosis angle and the FMS composite score in females with hyperlordosis. However, there was a negative correlation between the McGill test and lordosis angle. Therefore, the McGill test can be regarded as an accurate assessment tool for identifying any core muscle endurance deficit in women with lumbar lordosis disorder. However, according to the findings, FMS had not sensitivity to predict the injury in individuals with lordosis deformity. Thus, it is recommended to employ other useful tools to identify any compensatory movement and detect the improper movement patterns in individuals with hyperlordosis as well.

## Data Availability

All data generated or analyzed during this study are included in this published article.

## List of abbreviations

FMS: Functional Movement Screen.
